# Mechanism Study on the Regulation of Intestinal Microecology by Hangover Liver‐Protecting Beverage for the Treatment of Alcoholic Liver Disease

**DOI:** 10.1002/fsn3.71435

**Published:** 2026-01-07

**Authors:** Wen Cai, Ping Wang, Ru Zhang, Limin Dong, Jiayi Li, Mingming Wang, Lin Wang, Xuekun Shao, Tianyuan Jing, Yanan Hu, Yi Wang, Cheng Wang, Tiefeng Sun, Haitao Du, Xiaoyan Ding

**Affiliations:** ^1^ School of Pharmaceutical Sciences Shandong University of Traditional Chinese Medicine Jinan China; ^2^ Shandong Academy of Chinese Medicine Jinan China; ^3^ Department of Pharmacy Jinan Hospital of Traditional Chinese Medicine Jinan China; ^4^ School of Pharmacy Shandong Modern College Jinan China

**Keywords:** alcoholic liver disease, food and medicine, intestinal flora, intestinal‐liver axis, natural products, short‐chain fatty acids

## Abstract

In this study, we systematically investigated the preventive and curative effects of food‐sourced hangover liver‐protecting beverage (HLB) against alcoholic liver disease (ALD) and their multi‐target mechanisms through molecular docking, 16S rDNA sequencing, and functional assessment of the hepatic‐intestinal axis. Based on the theory of medicinal food harmony (MFH), 121 potential active components were identified from six foods, including *Hovenia dulcis* , chicory, and umeboshi, by UPLC‐QTOF‐MS/MS technology. HLB characteristic active components such as quercetin, catechin, and ferulic acid were verified through molecular docking. In vivo experiments showed that HLB significantly reduced serum LPS, ALT, AST, TC, and TG levels, upregulated the expression of intestinal tight junction protein (ZO‐1/Occludin), and inhibited the activation of the TLR4 (Toll‐like receptor 4)/NF‐κB/NLRP3 inflammatory axis in ALD model mice. Meanwhile, HLB restored the homeostasis of intestinal flora and the metabolism of short‐chain fatty acids (SCFAs), confirming that it synergistically ameliorated liver injury through the “flora‐SCFAs‐intestinal barrier” axis. This study reveals the molecular basis for the regulation of ALD by food‐based natural ingredients through liver – gut interactions and provides theoretical and practical basis for the development of functional beverages with both safety and multi‐target effects.

## Graphical abstract

Technical Roadmap for HLB‐Mediated ALD Therapy.

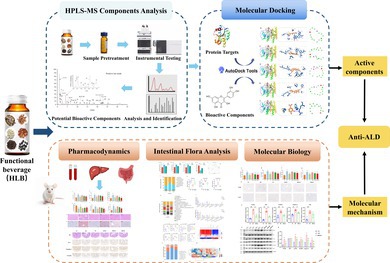

**Abbreviations**
ALD: alcoholic liver disease
ALT: alanine aminotransferase
AST: aspartate aminotransferase
GSH: glutathione
IL‐1β: interleukin‐1β
IL‐6: interleukin‐6
LPS: lipopolysaccharide
MDA: malondialdehyde
ROS: reactive oxygen species
RT‐qPCR: quantitative reverse transcription PCR
SCFAs: short‐chain fatty acids
TC: total cholesterol
TG: triglyceride
TLR4: toll‐like receptor 4
TNF‐α: tumor necrosis factor‐α

## 1 Introduction

Alcoholic liver disease (ALD) due to alcohol abuse is an important contributor to the global burden of liver disease (Udoh et al. 2015), with a pathologic process ranging from steatosis and inflammation to hepatic fibrosis, which is strongly associated with imbalances in host metabolic homeostasis and disturbances in the intestinal flora (Dukić et al. 2023; Mackowiak et al. 2024). The intestinal barrier dysfunction induced during alcohol metabolism leads to the migration of colony metabolites such as lipopolysaccharide (LPS) to the liver via the portal system, activating the TLR4/NF‐κB signaling axis and inducing increased levels of IL‐1β and the synthesis of NLRP3, further exacerbating ALD (Liu et al. 2022; Choudhury et al. 2020). Data from the World Health Organization show that approximately 3 million people die globally each year due to alcohol abuse, with ALD causing a significant proportion of liver failure (GBD 2016 Alcohol Collaborators 2018; Åberg et al. 2024). Existing therapies rely on alcohol cessation and pharmacologic interventions (e.g., glucocorticoids), but suffer from target limitations, long‐term side effects, and low patient compliance (Mackowiak et al. 2024), and safe and sustainable dietary intervention strategies are urgently needed.

Dietary beverages centered on natural plant components have gradually become a hotspot in ALD prevention and treatment research due to their multi‐component synergistic effect and low toxicity characteristics. *Hovenia dulcis* , chicory, *Pueraria lobata* , etc. are not only used as food for health care, but also have broad application prospects in disease prevention and treatment, with significant effects in lowering blood lipids (Perović et al. 2021), resisting alcoholic liver injury (Qiu et al. 2019), promoting bile acid secretion, and acting as antioxidants (Ji et al. 2020), among others. The food source detoxification and liver‐protecting beverage (HLB for short) developed in this study is based on *Hovenia dulcis* , *Pueraria lobata* , and six other plant raw materials, and is rich in quercetin, catechins, kudzu, and chlorogenic acid, among other active ingredients, which are known for their ability to enhance liver antioxidant capacity by targeting the liver‐intestinal axis through multiple pathways (Li et al. 2023), downregulate the expression of genes and proteins of inflammatory factors (Zhu et al. 2023), and regulate the ecological imbalance of gut microbiota and its metabolites (Qu et al. 2022) etc., to exert hepatoprotective effects. Unlike single‐target drugs for ALD, HLB utilizes multi‐component synergistic effects that may synchronize responses to oxidative damage, inflammatory cascades, and metabolic disorders to provide a holistic therapeutic solution. Traditional drugs such as glucocorticosteroids and metadoxine (Verma et al. 2024) can provide short‐term symptomatic relief, but there are problems such as a single target, metabolic burden, and toxic side effects of long‐term use. In contrast, long‐term consumption of food‐derived beverages has significant efficacy in the prevention and treatment of ALD (Tadokoro et al. 2023), including enhanced ethanol detoxification and hepatoprotection, and is safe, with high patient compliance and no significant side effects, and is now becoming a new direction in the treatment of ALD.

In recent years, research strategies to modulate the composition of intestinal flora to combat ALD based on bioactive ingredients from food sources have received much attention. However, the role of natural beverages with detoxification and hepatoprotective properties (HLBs) in this regard is still not fully understood. Therefore, the present study was carried out to identify and screen the effective active ingredients in HLB by UPLC‐QTOF‐MS/MS and molecular docking techniques to clarify the material basis of their medicinal effects. An ALD mouse model was constructed by alcohol gavage to systematically assess the effects of HLB on liver injury markers, inflammatory factors, and histopathological changes. Combined with 16S rDNA sequencing analysis, it reveals its therapeutic effect on alcohol‐induced imbalance of gut microbiota as well as intestinal barrier dysfunction. In‐depth understanding of the nutritional potential of HLB in the prevention and treatment of ALD provides a theoretical and practical basis for the regulation of the “gut–liver” axis in food‐based interventions for ALD.

## 2 Materials and Methods

### 2.1 Preparation of Hangover Liver‐Protecting Beverage

The MFHs required for the preparation of HLB, *Hovenia dulcis* , umeboshi, hawthorn, chicory, sorrel, and *Pueraria lobata* were purchased from Jinan Jianlian Shengjia Traditional Chinese Medicine Company Limited (Jinan, China). The above MFHs were pulverized at high speed and decocted for 1 h at a material‐water ratio of 1:10 (w/v) and extracted twice. The extracts were combined and concentrated to prepare a final concentration of 1 g/mL of HLB, which was stored in a refrigerator at 4°C for spare use.

### 2.2 UPLC‐QE‐MS/MS

Vanquish UHPLC and Q‐Exactive Orbitrap Plus (Thermo Fisher, Waltham, MA, USA) were used to analyze and identify the active ingredients of the antidepressant and liver‐protective beverages. The Hypersil GOLD VANQUISH C18 UHPLC (1.9 μm 100 × 2.1 mm) column was operated at 35°C. MS analysis was performed in positive and negative ion mode. Raw data were analyzed and processed by Xcalibur Qual Browser (Thermo Fisher, Waltham, MA, USA) software.

### 2.3 Molecular Docking

We obtained the 3D structures of the key active ingredients as SDF format files from PubChem, and the 3D structures of the TLR4 receptor proteins were downloaded from the PDB database in PDB format. These structure files were imported into AutoDock Vina for molecular docking and calculation of binding energies. Docking results are visualized through PyMOL.

### 2.4 Animal Experiment Design

Male C57BL/6 mice weighing 20–22 g, 8 weeks old, were purchased from Jinan Pengyue Laboratory Animal Breeding Co. Ltd. (Jinan, China), Animal License No. SCXK (LU) 20,220,006, and this experiment was approved by the Ethics Committee of the Shandong Institute of Traditional Chinese Medicine (Jinan, China) (Grant No. SDZYY20211001002) and kept in the SPF‐class laboratory of the Animal Experiment Center of the Shandong Institute of Traditional Chinese Medicine. This experiment was approved by the Ethics Committee of Shandong Institute of Traditional Chinese Medicine (Jinan, China) (Grant No. SDZYY20211001002). Feeding conditions: temperature in the range of 22°C ± 1°C, humidity 55% ± 5%, light and dark cycles of 12 h each, administration of sterilized feed and sterilized water. After acclimatization feeding, the mice were randomly divided into normal group (NC), MC group (MC), SF group (positive drug group, silymarin), low‐dose group of detoxification and liver‐protecting drink (LHLB), middle‐dose group of detoxification and liver‐protecting drink (MHLB), and high‐dose group of detoxification and liver‐protecting drink (HHLB), with 7 mice in each group. Mice in the MC, SF, and HLB groups were given liquid chow containing 5% anhydrous ethanol for 10 consecutive days (Bio‐Serv, Frenchtown, NJ, USA), and mice in the NC group were given regular liquid chow without anhydrous ethanol (Bio‐Serv, Frenchtown, NJ, USA). Along with liquid chow, mice were gavaged with 0.9% saline, silymarin (40 mg/kg) (Hui Zhi Pharmaceutical Co. Ltd., Dalian, China), and different doses of antidepressant and liver‐protecting beverages daily for 20 days. The equivalent dose of the drug was calculated based on the body surface area of human and animals and the dose of HLB in mice was 2.665, 5.33 and 10.66 g/kg for the low, medium and high groups, respectively.

### 2.5 Histopathologic Testing

To evaluate the efficacy of HLB in the treatment of ALD, histopathological changes were observed in the liver and colon of mice. Liver and colon tissue specimens were collected, rinsed with PBS, and fixed in 10% formalin. In order to observe the cell structure, the sections were stained with hematoxylin and eosin (HE), and the pathological changes of mouse liver and colon tissues were observed under the light microscope; oil red O staining was used to observe the degree of hepatic steatosis under the light microscope.

### 2.6 Measurement of Lipid‐Related Indices in Serum

The serum to be measured was prepared by centrifugation at 3000 r/min for 15 min after the whole blood was allowed to stand for 2 h. Determination of lipid accumulation‐related markers using assay kits: These included a triglyceride (TG) assay kit, a total cholesterol (TC) quantitative assay kit, as well as an ALT assay kit and an AST assay kit (all purchased from Nanjing Jiancheng Bioengineering Institute, Nanjing, China).

### 2.7 Enzyme‐Linked Immunoassay

The liver tissue homogenate was centrifuged in pre‐cooled saline with a mass to volume ratio of 1:9 at 3000 r/min for 15 min at 4°C to obtain the liver homogenate supernatant. TNF‐α, IL‐1β, and IL‐6 inflammatory factors in liver tissues and LPS in serum were detected using enzyme immunoassay kits (Shanghai Enzymatic Biotechnology Co. Ltd., Shanghai, China).

### 2.8 Immunohistochemical Tests

Paraffin sections were dehydrated for antigen repair, sections were placed in 3% hydrogen peroxide to block endogenous peroxidase, rabbit serum was closed and shaken dry, and primary antibody was incubated at 4°C overnight. After washing, shake dry and add the corresponding secondary antibody to incubate at room temperature; after washing, add DAB to develop the color, hematoxylin re‐staining, washing, differentiation, and return to the blue, after dehydration and sealing, and read the results under the microscope.

### 2.9 Real‐Time Fluorescence Quantitative PCR and Western Blot

Total RNA was extracted from mouse liver tissues by an extraction kit (Wuhan Jewell Biotechnology Co. Ltd., Hubei, China) and cDNA was synthesized by a reverse transcription system according to the instructions of SweScript All‐in‐One RT SuperMix for qPCR kit (Wuhan Jewell Biotechnology Co. Ltd.). Amplification results were detected using a real‐time fluorescence quantitative PCR instrument (Applied Biosystems Inc., Waltham, MA, USA) according to the 2 × Universal Blue SYBR Green qPCR Master Mix kit (Wuhan Javelin Biotechnology Co. Ltd., Hubei, China). The primer sequences are shown in Table S1.

Total proteins were extracted from liver tissues using RIPA lysis buffer, and the protein concentration was determined with the BCA Protein Assay Kit (Shanghai Yamei Biomedical Technology Co. Ltd., Shanghai, China). Proteins from different sample groups were separated by SDS‐PAGE gel and then transferred onto PVDF microporous membranes. The transferred membranes were rinsed rapidly three times with 1 × TBST buffer, followed by blocking with non‐fat milk powder on a decolorizing shaker at room temperature for 5 min. Primary antibodies were added for incubation at 4°C overnight, and after quick rinsing with 1 × TBST buffer, the membranes were incubated with secondary antibodies at room temperature for 30 min, followed by additional rinsing with 1 × TBST buffer. Finally, visualization was performed using ECL developing solution (Shanghai Yamei Biomedical Technology Co. Ltd., Shanghai, China), and gray value analysis was conducted with Image J software.

### 2.10 High‐Throughput Sequencing of Intestinal Flora

The colon contents of each group of mice were collected for DNA extraction after dissection. PCR amplification of the V3‐V4 variable region of the 16S rRNA gene was carried out with upstream primer 338F (5′‐ACTCCTACGGGGAGGCAGCAG‐3′) and downstream primer 806R (5′‐GGACTACHVGGGTWTCTAAT‐3′), which carry the Barcode sequence. The products were purified by AxyPrep DNA Gel Extraction Kit, quantified by Quantus Fluorometer, and then sequenced bipartite by Illumina Miseq PE300 sequencer. The raw sequences were quality controlled by fastp (https://github.com/OpenGene/fastp,v0.19.6) and spliced by FLASH (http://www.cbcb.umd.edu/software/flash,v1.2.11), and then clustered by UPARSE (http://clustering.com/uparse/, v7.1) at 97% similarity and chimeras were removed by OTU at 97% similarity and chimeras, followed by leveling of the sample sequence numbers to eliminate the effect of sequencing depth, and with the help of RDP classifier (http://rdp.cme.msu.edu/, v2.11) to compare the Silva 16S rRNA database (v138) to complete species annotation, and PICRUSt2 (v2.2.0) was utilized for 16S functional prediction analysis.

### 2.11 Determination of Short‐Chain Fatty Acid Content

Fecal samples were processed by cryomilling combined with ultrasound‐assisted extraction, and after low‐temperature high‐speed centrifugation and liquid–liquid extraction with n‐butanol, short‐chain fatty acids (SCFAs) were quantified using an Agilent 8890B‐7000D gas chromatography–mass spectrometry instrument (Agilent, Santa Clara, CA, USA).

### 2.12 Data Analysis

SPSS 24.0 software was used to process the experimental data, which were expressed as x ± s. One‐way ANOVA was used for comparison between multiple groups, and *p* < 0.05 indicated that the difference was statistically significant.

## 3 Results

### 3.1 Main Ingredients in Hangover Liver‐Protecting Beverage

**FIGURE 1:**
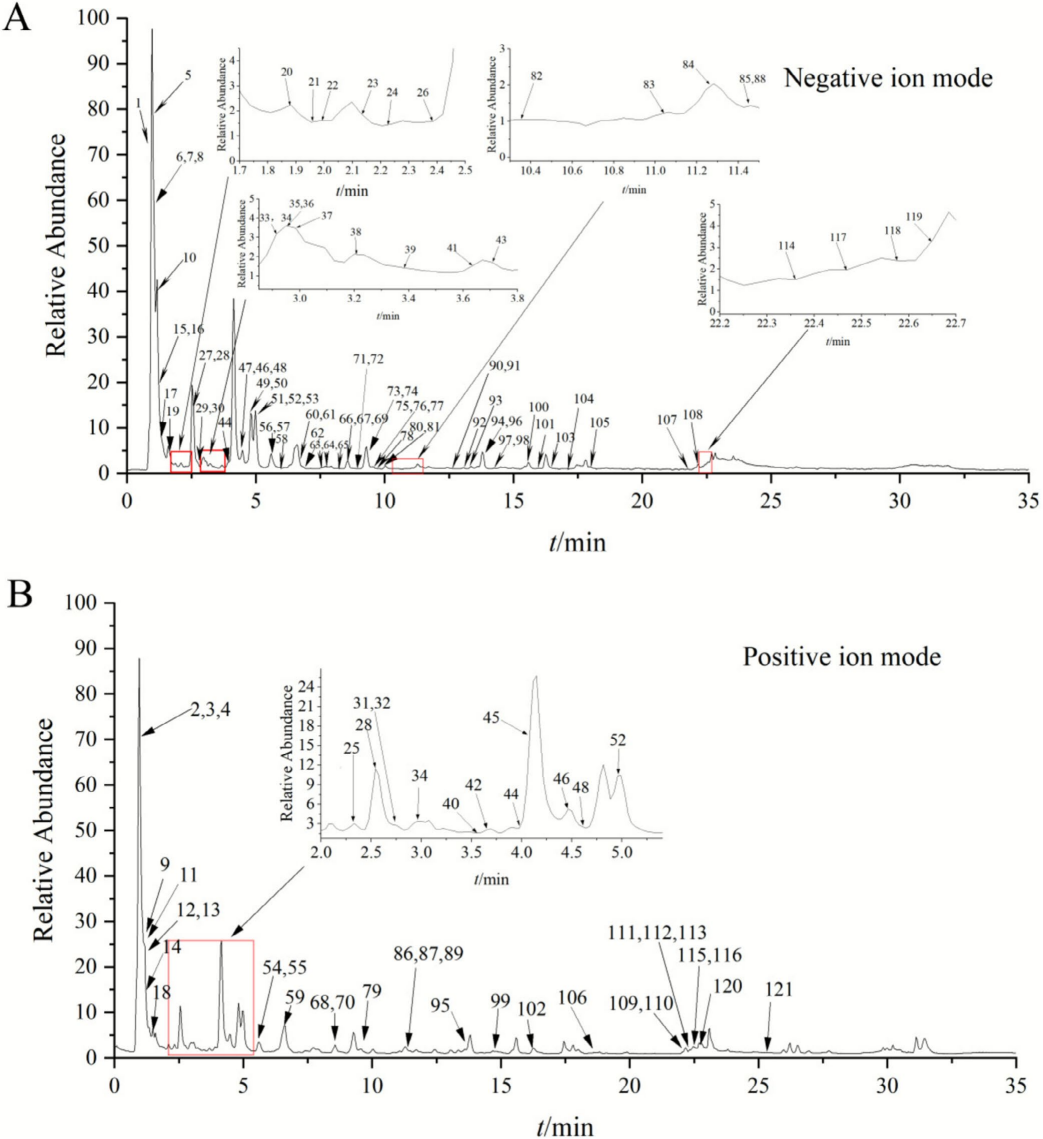
Results of LC–MS analysis in negative ion (A) and positive ion (B) mode.

**FIGURE 2:**
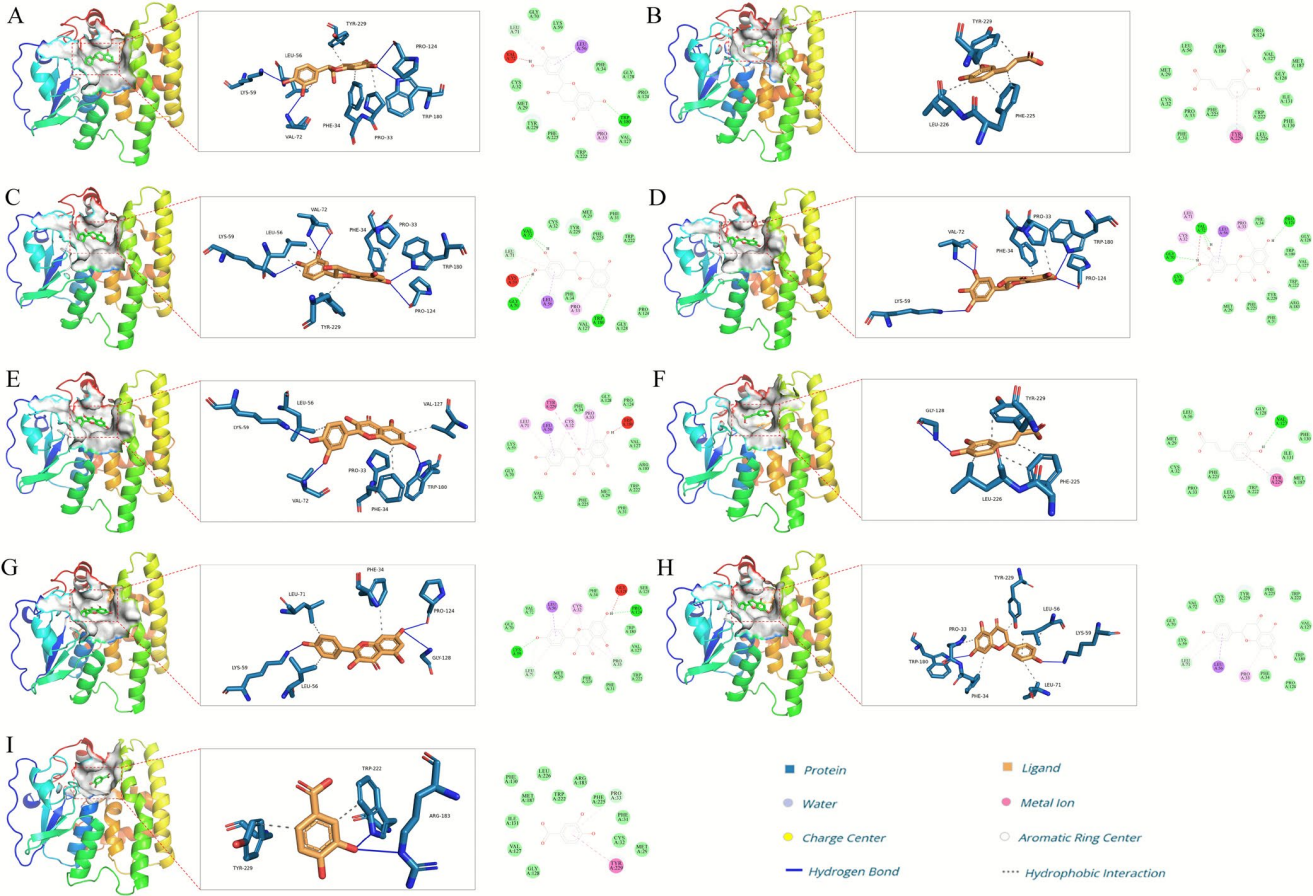
The results of molecular docking: (A) Epicatechin, (B) Quercetin, (C) Dihydromyricetin, (D) Catechin, (E) Kaempferol, (F) Naringin, (G) Ferulic acid, (H) Caffeic acid, and (I) Protocatechuic acid.

The chemical constituents identified from this alcohol‐ and liver‐protecting beverage using UPLC‐QE‐MS/MS technique included flavonoids, amino acids, lignans, terpenoids, coumarins, organic acids and their derivatives. After self‐constructed library search and standard comparison, excluding duplicates, a total of 121 compounds were identified (Table S2), and the total ion flow diagram is shown in Figure 1. There are 59 flavonoids, 8 amino acids, 23 organic acids and their derivatives, 1 lignan, 16 terpenoids, 5 coumarins, and 9 other compounds. Combining the active ingredients of the six herbs reported to exert medicinal effects in the relevant literature and comparing them with the control, the candidate active ingredients of HLB were finally integrated to obtain the candidate active ingredients, including quercetin, catechin, epicatechin, naringenin, dihydromyricetin, kaempferol, ferulic acid, protocatechuic acid, and caffeic acid (Figure S1). It was hypothesized that these nine compounds might be key components for the treatment of alcohol‐related liver disease. The molecular docking results showed that the binding energy threshold of each active ingredient to TLR4 protein was less than −5 kcal/mol, indicating good binding activity (Figure 2 and Table S3). HLB may act on TLR4 proteins through these active ingredients to inhibit the activation of the TLR4/NF‐κB signaling pathway and downregulate the release of pathway‐mediated pro‐inflammatory factors (e.g., TNF‐α, IL‐6), thus intervening in the ALD pathological process in a multi‐targeted manner.

### 3.2 Hangover Liver‐Protecting Beverage Ameliorate Ethanol Liver Injury by Inhibiting Lipid Accumulation

**FIGURE 3:**
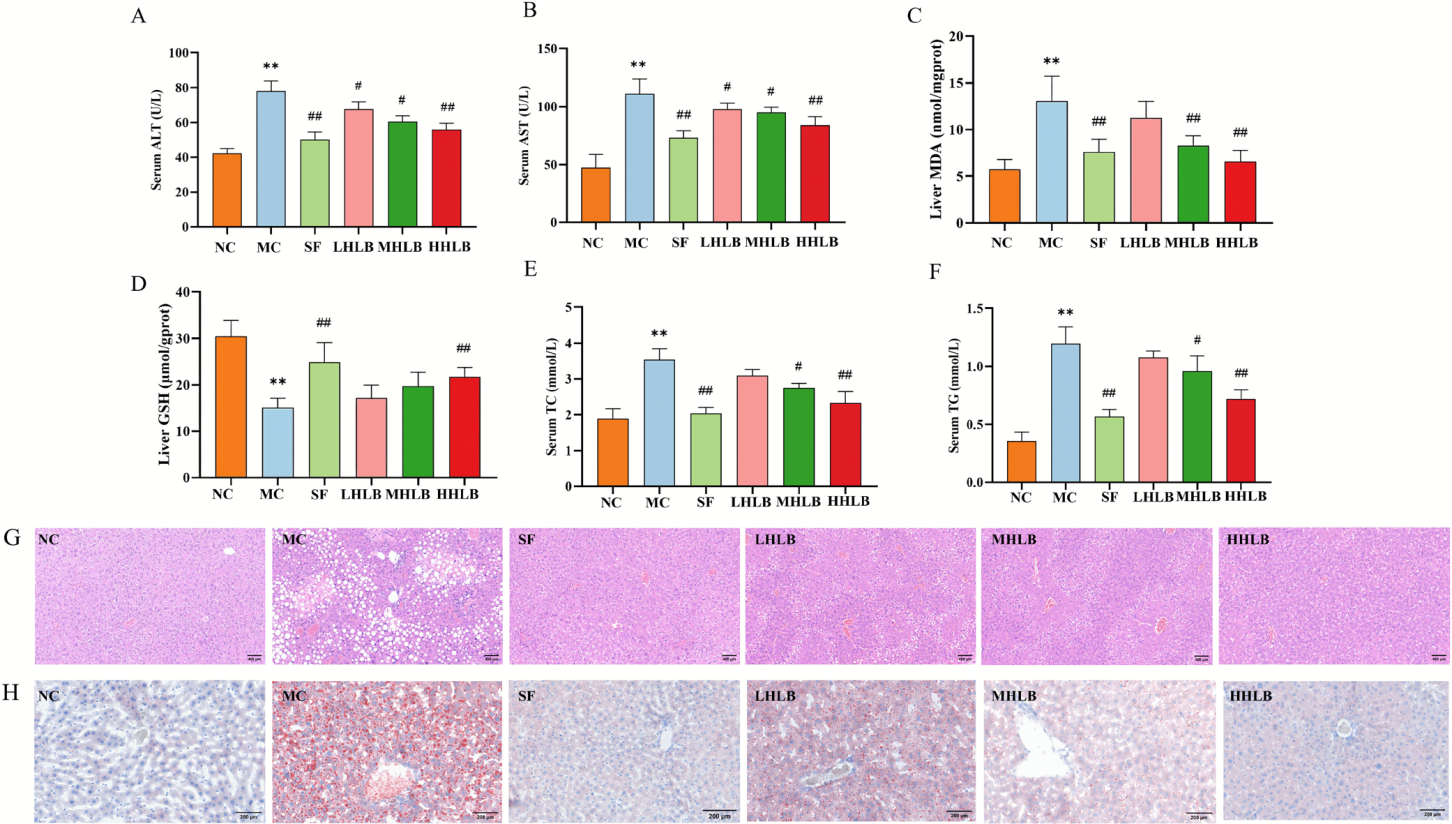
Alcohol‐ and liver‐protective beverages attenuate alcohol‐induced liver injury: (A) Serum ALT; (B) Serum AST; (C) Liver MDA; (D) Liver GSH; (E) Serum TC level; (F) Serum TG level; (G) HE staining to observe histopathological changes in the liver (×200); (H) Liver oil red O staining (×400).

To evaluate the hepatoprotective effect of HLB in ALD model mice, serum transaminase levels and histopathological changes in the liver were systematically analyzed. Biochemical assays showed that serum ALT and AST activities were significantly elevated in the model group (*p* < 0.01, Figure 3A,B), suggesting severe hepatic injury, as well as an increase in hepatic lipid peroxidation products (MDA) and depletion of reduced glutathione (GSH), indicating an imbalance in redox homeostasis (Figure 3C,D). HLB intervention dose‐dependently inhibited transaminase activity and oxidative stress levels. Histological observation showed (H&E staining, Figure 3G) that the liver lobules in the control group were structurally intact, whereas the model group showed microvesicular steatosis, inflammatory cell infiltration, and edema; the HLB‐treated group (especially the high‐dose group) significantly ameliorated the above pathologic injuries.

Hepatic steatosis (a core pathological feature of ALD) was assessed by lipid analysis and oil red O staining. Serum total cholesterol (TC) and triglyceride (TG) levels were significantly elevated in the model group (*p* < 0.01, Figure 3E,F), and lipid levels were reduced in the HLB‐treated group in a dose‐dependent manner. Oil red O staining (Figure 3H) showed diffuse lipid droplet deposition in the hepatocytes of the model group, whereas lipid accumulation was significantly reduced in the HLB intervention group, with the best improvement in the high‐dose group, where hepatocyte morphology was normalized. These results indicate that HLB significantly alleviated ALD and its concomitant steatosis pathological process by inhibiting hepatic lipid accumulation and attenuating ethanol‐induced oxidative stress and inflammatory response.

### 3.3 Hangover Liver‐Protecting Beverage Reduce Intestinal Damage Induced by Alcohol Intake

**FIGURE 4:**
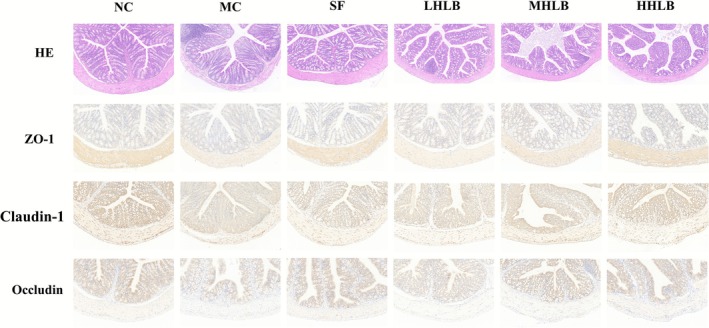
Hangover liver‐protecting beverage attenuates alcohol‐induced intestinal damage. Changes in colonic histopathology observed by HE staining and changes in colonic classified ligand protein expression observed by IHC staining (×100).

The effect of HLB on the gut was assessed by H&E staining of colon tissue. The results showed that alcohol exposure led to epithelial damage, increased vacuoles in the lamina propria, decreased cuprocytes, and inflammatory infiltration in the colonic mucosa of mice in the model group, while HLB intervention dose‐dependently improved the above pathologic changes, with the best improvement in the high‐dose group (Figure 4). Compared with normal mice, the expression level of core structural proteins of the intestinal mucosal barrier was downregulated, and intestinal epithelial permeability was increased in MC group mice. In contrast, HLB was able to significantly upregulate the expression of alcohol‐inhibited ZO‐1, Claudin‐1, and Occludin, repair the integrity of the intestinal barrier, reduce the intestinal‐derived endotoxin translocation, and modulate the hepatic‐intestinal axis to alleviate alcohol‐induced liver injury.

### 3.4 Hangover Liver‐Protecting Beverage Ameliorate Alcoholic Liver Disease by Inhibiting the TLR4/NF‐κB Pathway

**FIGURE 5:**
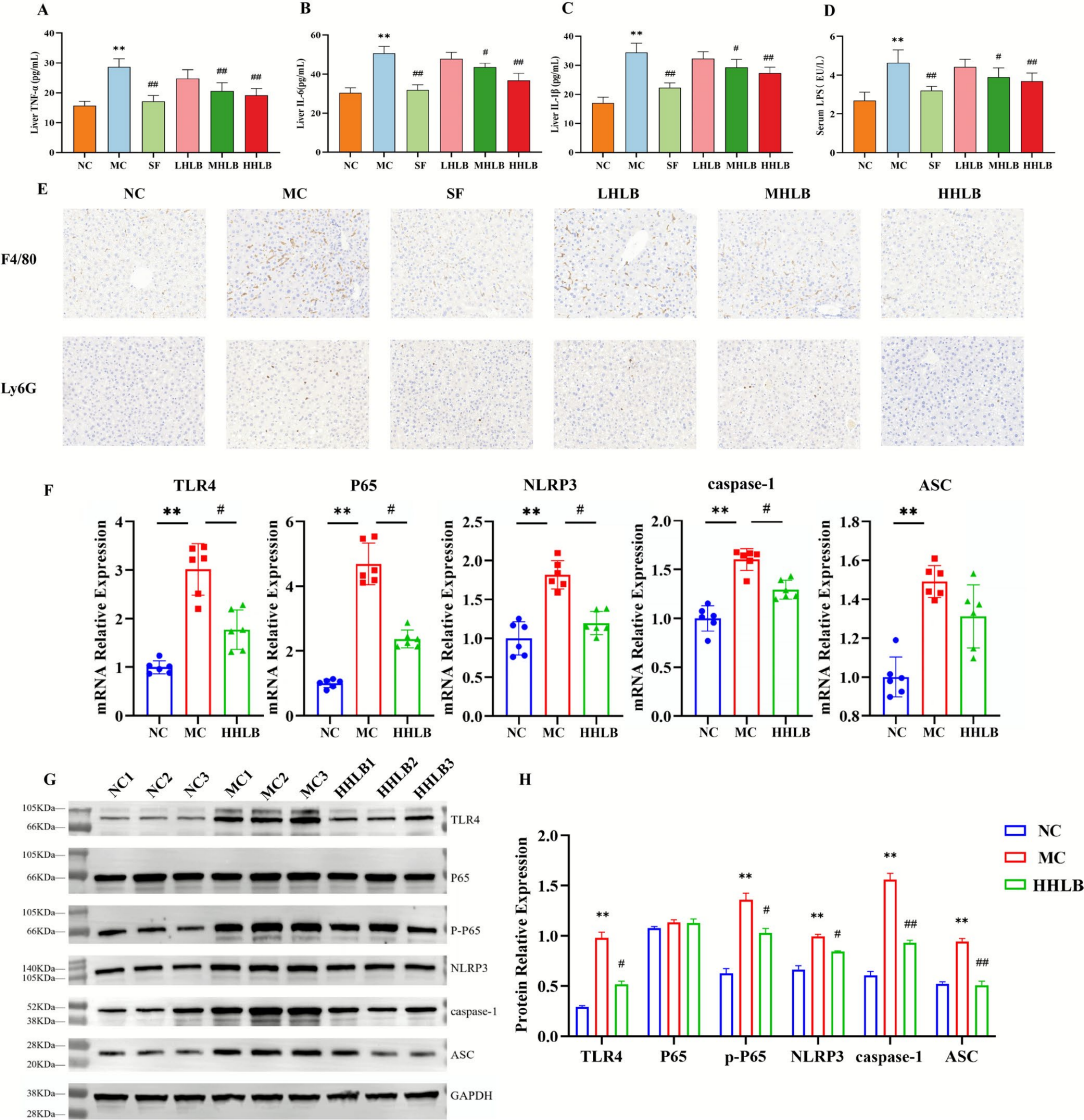
Inhibition of TLR4/NF‐κB pathway by antidepressant and hepatoprotective beverages ameliorates alcoholic liver disease: (A–E) Liver inflammatory factors and serum endotoxin levels. (A) TNF‐α; (B) IL‐6; (C) IL‐1β; (D) LPS; (E) Immunohistochemical staining of F4/80 and Ly6G (×200); (F) Relative mRNA expression levels of TLR4, NF‐κB p65, NLRP3, ASC and caspase‐1; (G, H) Relative expression levels of TLR4/NF‐κB/NLRP3 pathway‐related proteins.

Serum LPS levels and hepatic TLR4/NF‐κB/NLRP3 inflammatory axis activation were significantly higher in the ALD model group of mice than in the normal group (NC) (*p* < 0.01). After the intervention of detoxifying and liver‐protecting beverages, the high‐dose group downregulated the gene and protein expressions of TLR4, NF‐κB p65 phosphorylation (P‐P65), and NLRP3 inflammatory vesicle (ASC/caspase‐1) (Figure 5F–H). Inhibited inflammatory signaling driven by intestinal‐derived endotoxin (LPS) (Figure 5E). Significantly reduced hepatic pro‐inflammatory factor (TNF‐α, IL‐6, IL‐1β) levels (Figure 5A–C). In the immunohistochemical results, blue color is the nucleus after hematoxylin staining, and brown color is the positive expression of target proteins. Macrophage (F4/80) and neutrophil (Ly6G) expression was significantly increased in the livers of the mice in the MC group, and the administration of ZJ reduced the infiltration of macrophages and neutrophils. This suggests that ZJ may attenuate alcohol‐induced liver inflammation by inhibiting the TLR4/NF‐κB pathway, attenuating macrophage M1‐type polarization, and inhibiting neutrophil infiltration in mouse liver.

### 3.5 Hangover Liver‐Protecting Beverage Improve Intestinal Flora Disorders Caused by Alcohol Intake

**FIGURE 6:**
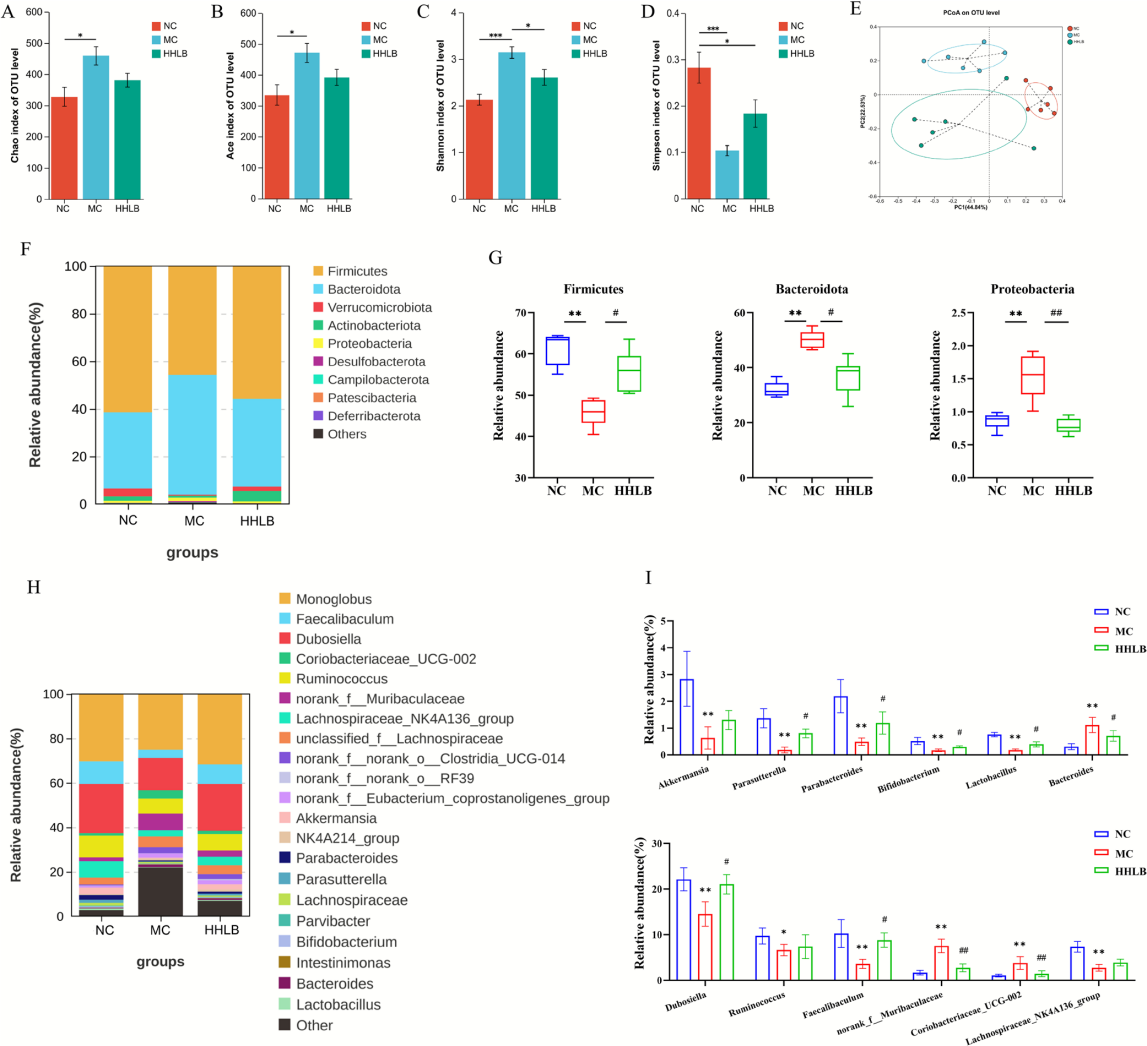
Hangover liver‐protecting beverage improves intestinal flora alpha diversity (A–E) and intestinal flora disorders (F–I): (A) Chao Index; (B) Ace Index; (C) Shannon Index; (D) Simpson Index; (E) PCoA analysis; (F) Distribution of intestinal flora at the phylum level; (G) Differential flora at the phylum level; (H) Distribution of intestinal flora at the genus level; (I) Differential flora at the genus level.

Alpha diversity analysis showed that alcohol intake significantly increased Chao, ACE (abundance index), and Shannon index (diversity index) and decreased Simpson index (*p* < 0.01 or *p* < 0.05, Figure 6A–D) in the intestinal flora of ALD model group (MC) mice, suggesting that alcohol disrupts the homeostasis of intestinal flora and induces ecological imbalance. The HLB intervention effectively inhibited the proliferation of conditionally pathogenic bacteria and restored the flora structure. PCA analysis (Figure 6E) showed that the samples in the HHLB group clustered more closely with the normal group (NC), suggesting that it significantly reversed alcohol‐induced dysbiosis and reshaped the balance of microbial‐host interactions.

Gate‐level analysis showed that the intestinal flora of mice in the ALD model group (MC) had significantly lower abundance of Firmicutes (*p* < 0.01) and higher abundance of Bacteroidota and Proteobacteria (Figure 6F,G), suggesting that alcohol exposure led to an imbalance in the bacterial flora and enrichment of potential pathogenic bacteria. The HLB intervention significantly reversed the above abnormal phylum proportions (*p* < 0.01 or *p* < 0.05). At the genus level, the abundance of SCFA‐producing bacteria in the MC group (e.g., Faecalibaculum, Dubosiella, Ruminococcus, Parabacteroide, Parasutterella, Lachnospiraceae_NK4A136_group Bifidobacterium bifidobacterium, Lactobacillus lactobacillus) and mucus‐degrading bacteria (Akkermansia) were significantly reduced in abundance, whereas pro‐inflammatory related genera (e.g., Bacteroides, norank_f__Muribaculaceae) were elevated in abundance (Figure 6H,I). HLB remodeled the functional network of intestinal flora and promoted SCFA synthesis and intestinal barrier repair by upregulating the abundance of beneficial commensal bacteria and inhibiting the proliferation of conditionally pathogenic bacteria.

### 3.6 Hangover Liver‐Protecting Beverage Restore Short‐Chain Fatty Acids in the Cecum

**FIGURE 7:**
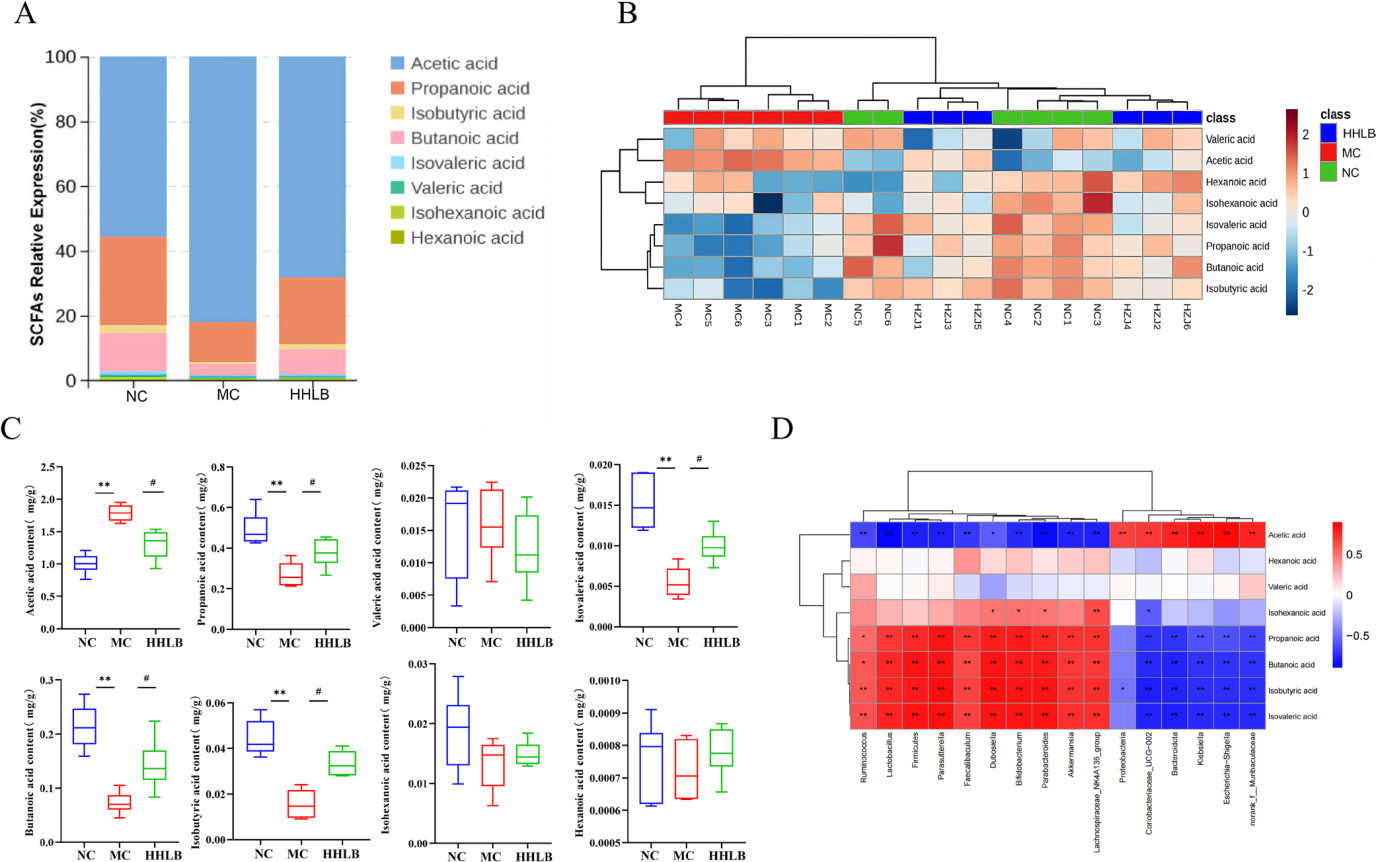
Effects of hangover liver‐protecting beverage on short‐chain fatty acid content in the cecum of mice: (A) Stacked plots of eight short‐chain fatty acids; (B) Heat maps; (C) Differences in short‐chain fatty acid content; (D) Correlation analysis of short‐chain fatty acids with differential bacteria.

Analysis of SCFAs showed that the SCFA species in the cecum contents of mice in all groups consisted mainly of acetic, propionic, and butyric acids. Acetic acid levels in the cecum of mice in the ALD model group were significantly higher (*p* < 0.01), whereas propionic acid, butyric acid, isobutyric acid, and isovaleric acid levels were significantly lower (*p* < 0.01), suggesting that alcohol interferes with the metabolic homeostasis of intestinal SCFAs (Figure 7A–C). After HLB intervention, the high‐dose group significantly reduced acetic acid levels (*p* < 0.05) and restored the synthesis of beneficial SCFAs such as propionic acid and butyric acid (*p* < 0.05). Thermogram and PCA analysis (Figure 7B) showed that the spectrum of SCFAs in the HLB group was close to that of the normal group (NC), confirming its function of remodeling the metabolism of the flora. Correlation analysis (Figure 7D) further revealed that HLB upregulated Firmicutes and SCFAs‐producing genera (e.g., Bifidobacterium bifidobacterium, Lactobacillus lactobacillus) were positively correlated with propionic acid and butyric acid (*p* < 0.05), whereas model group‐enriched Bacteroidota and pro‐inflammatory genera (e.g., Bacteroides) were positively correlated with acetic acid and negatively correlated with beneficial SCFAs, suggesting that HLB modulates the metabolic‐inflammatory network of the hepatic‐gut axis through colony‐SCFAs interactions. The present study demonstrated that the Lipoprotective Drink synergistically improved the ALD process by targeting the enrichment of SCFAs‐producing flora, inhibiting the abnormal accumulation of acetic acid, and restoring the biosynthesis of beneficial SCFAs (propionic acid and butyric acid).

## 4 Discussion

Functional foods have gradually become a research hotspot in the field of ALD prevention and treatment due to their natural origin, multi‐target regulation, and high safety features. HLB, as a functional dietary formula, was identified by mass spectrometry to be rich in flavonoids (naringenin, kaempferol, quercetin, etc.) and organic acids (ferulic acid, caffeic acid, etc.) active ingredients. These bioactive substances exert hepatoprotective effects by synergistically inhibiting the release of pro‐inflammatory factors, regulating the homeostasis of lipid metabolism, and improving the dysbiosis of intestinal flora (Luo et al. 2022; Cao, Yue, et al. 2023; Mu et al. 2021; Hong et al. 2025), and have the potential to be used as a food‐borne intervention agent in the long term. HLB highlights the multidimensional intervention advantages of functional foods through the “liver‐intestinal axis” mechanism: on the one hand, it improves the metabolism of intestinal toxins through the reconstruction of intestinal flora homeostasis, and on the other hand, it acts directly on the hepatic cells through the portal system to form a bidirectional regulatory network. This multi‐pathway synergistic mode of action based on natural ingredients not only avoids the toxic side effects of chemical drugs on the body but also provides a new way of thinking to develop ALD intervention strategies with both nutritional functions and therapeutic effects.

In the assessment of the pathological process of ALD, serum ALT/AST levels, as the classical markers for the disruption of hepatocyte membrane integrity, are abnormally elevated to directly reflect the degree of hepatic parenchymal injury (Shuwen and Kefeng 2022). The accumulation of TG/TC, on the other hand, is closely related to the inhibition of fatty acid β‐oxidation and the promotion of lipid de novo by alcohol, and is a core indicator for the assessment of lipid metabolism disorders (Niu et al. 2022). The results of the present study showed a significant increase in the above indexes in the model group, which is consistent with the mechanism reported in the literature (Leung and Nieto 2013; Wang et al. 2023) that alcohol intake in large quantities leads to CYP2E1 overactivation triggering a ROS burst, which in turn leads to mitochondrial dysfunction. Notably, depletion of GSH, a key intracellular antioxidant, and accumulation of the lipid peroxidation end product MDA confirm the pivotal role of oxidative stress in ALD progression (Harjumäki et al. 2021; Attal et al. 2022). The indicators were significantly improved after HLB intervention in a dose‐dependent manner. HLB suppresses the hepatic accumulation of total TC and TG. This reduces substrates available for lipid peroxidation, which in turn downregulates the level of MDA—a well‐established marker of oxidative stress. Concurrently, it upregulates the content of GSH, a critical endogenous antioxidant. Together, these effects synergistically alleviate alcohol‐induced hepatic oxidative stress damage. HE staining results showed structural remodeling of hepatic lobules and a reduction in the area of oil red O‐stained lipid droplets, confirming its ability to attenuate alcohol‐induced disorders of hepatic lipid metabolism and regulate the balance of the antioxidant system to attenuate alcohol‐induced hepatic injury. The results of this study suggest a dual protection of hepatocyte structure and function by functional foods, which may go beyond mere antioxidant compensation.

Alcohol‐induced hepatic inflammation involves a variety of complex mechanisms, the core mechanism of which lies in LPS translocation (Shuwen and Kefeng 2022; Chen et al. 2022; An et al. 2022) and its triggered hyperactivation of the TLR4/NF‐κB/NLRP3 inflammatory axis. In addition, increased expression of F4/80 and Ly6G, which are specific markers for macrophages and neutrophils, respectively, is closely associated with alcohol‐induced inflammatory liver injury (Wu, Fan, McMullen, et al. 2023; Wang et al. 2020; Hardesty et al. 2023). In the present study, we found that serum LPS levels were elevated in the ALD model group compared with the normal group, which drove the burst release of pro‐inflammatory factors such as TNF‐α and IL‐1β through activation of the hepatic TLR4 receptor, which in turn induced the phosphorylation of NF‐κB p65 and the assembly of NLRP3 inflammatory vesicles. This pathological process forms a vicious circle with macrophage M1‐type polarization and neutrophil activation, leading to a continuous deterioration of the inflammatory microenvironment of liver tissue (Bukong et al. 2018; Kawaratani et al. 2013). Of interest, HLB intervention can break this cycle by downregulating key nodes of the TLR4/NF‐κB pathway and inhibiting NLRP3 inflammatory vesicle activation, with a strength of action comparable to that of the positive drug silymarin. It also reduced LPS translocation dosage in a dose‐dependent manner, blocking inflammatory triggers at the source. F4/80 and Ly6G positive expression was also significantly reduced in liver tissues, demonstrating that HLB can modulate immune cell phenotype, reduce macrophage infiltration and neutrophil density, and ameliorate alcohol‐induced hepatic inflammatory response.

In addition to these direct effects, the modulation of the intestinal flora structure by HLB is also one of its mechanisms in the treatment of ALD. The progression of ALD is closely related to intestinal barrier disruption and flora‐metabolism disorders (Wang et al. 2021; Szabo 2015; Wu, Fan, Miyata, et al. 2023). Chronic alcohol exposure leads to a significant reduction in the expression of intestinal tight junction proteins (Claudin‐1, ZO‐1, Occludin), triggering leaky gut and promoting LPS translocation (Shao et al. 2018; Forsyth et al. 2014; Zhao et al. 2021). This suggests that HLB may promote the restoration of the intestinal epithelial mucosal barrier, a key factor in maintaining intestinal homeostasis and alleviating the progression of ALD inflammation. Notably, HLB may achieve intervention through the bidirectional regulation of intestinal flora by reestablishing probiotics and suppressing harmful bacteria. Faecalibaculum, Dubosiella, Akkermansia, Bifidobacterium, Lactobacillus, Ruminococcus, Parabacteroide, and Parasutterella in MC group mice, Lachnospiraceae_NK4A136_group, the relative abundance of beneficial bacteria decreased significantly, while the abundance of harmful bacteria such as norank_f__Muribaculaceae, Bacteroides, and Coriobacteriaceae_UCG‐002 increased significantly. After the HLB intervention, the composition of gut flora at the genus level was significantly improved. Among them, Bifidobacterium, as the predominant probiotic in the gut, is involved in triglyceride and bile acid metabolism, which is closely related to liver health (Hizo and Rampelotto 2023; Cao, Jin, et al. 2023). Recent studies have shown that Bifidobacterium has a favorable therapeutic effect on alcoholic liver injury, as evidenced by the reduction of hepatic inflammation and oxidative stress, and the improvement of alcohol‐induced gut microbial disorders and intestinal inflammation (He et al. 2022; Ehrlich et al. 2020; Samara et al. 2022). In addition, the relative abundance of Lactobacillus can reflect the health status of the host to a certain extent, and it has been shown that Lactobacillus can significantly inhibit alcohol‐induced steatosis and liver injury, restore the homeostasis of intestinal flora, and improve the intestinal barrier function (Ding et al. 2022). Akkermansia, which colonizes the intestinal mucus, can regulate intestinal permeability and improve inflammatory responses by degrading mucins in the intestinal mucosa (Sparfel et al. 2024). Several studies have shown a positive correlation between Coriobacteriaceae_UCG‐002 and body fat levels, in addition to the ability of this bacterium to produce harmful substances such as phenol and p‐cresol, which increases intestinal permeability and promotes intestinal inflammatory responses (Yu et al. 2023; Wang et al. 2022). Therefore, we hypothesized that HLB could enhance the intestinal barrier function and attenuate the inflammatory response in ALD by modulating the composition of the intestinal flora. Compared with pure anti‐inflammatory drugs, HLB synchronizes intestinal ecological repair and metabolic regulation through food‐borne components, providing a strategy more in line with physiological regulatory characteristics for ALD intervention.

SCFAs, metabolites of intestinal flora, are key signaling molecules in the “liver‐gut axis” (Qian et al. 2022; Ganesan et al. 2024), which can directly or indirectly affect the physiological functions and pathological manifestations of the liver. Chronic alcohol intake triggered metabolic disorders in SCFAs, with decreased levels of beneficial metabolites, such as butyric acid and propionic acid, as well as an abnormal accumulation of acetic acid in mice in the MC group (Martino et al. 2022; Flint 2016; Reichardt et al. 2014). This imbalance exacerbates liver injury through a dual mechanism: firstly, it disrupts the intestinal barrier, with butyric acid deficiency leading to downregulation of the expression of intestinal tight junction proteins (Claudin‐1, ZO‐1, Occludin) and an increase in intestinal epithelial permeability, which promotes LPS translocation and activation of the hepatic TLR4/NF‐κB inflammatory pathway (Ji et al. 2016; Zheng et al. 2017; Wang et al. 2012); and secondly, it leads to a dysregulated metabolism: propionic acid depletion impairs its regulation of the PPARγ pathway and exacerbates hepatic lipid accumulation (Xu et al. 2022; Jiang et al. 2020). HLB intervention restored the metabolic homeostasis of SCFAs by remodeling the bacterial colony structure (e.g., Bifidobacterium, Lactobacillus), in which butyric acid enhanced the intestinal barrier function by activating the GPR109A receptor, propionic acid modulated the lipid metabolism of hepatocytes through portal transport, and the retuning of the acetic acid/propionic acid ratio effectively inhibited the pro‐inflammatory effect of acetic acid and restored the intestinal barrier function that also restored the intestinal barrier function and alleviated the ALD effect. This cascade of “colony remodeling‐SCFAs recovery‐barrier repair”, combined with the inhibition of the TLR4/NF‐κB pathway discovered in the previous study, illustrates the multi‐targeted nature of ALD intervention by HLB through the “liver‐intestinal co‐therapy”. In addition, food therapy can be seamlessly integrated into lifestyles through daily diets, combining both preventive and therapeutic functions, and is particularly suitable for the long‐term management of chronic diseases such as ALD.

## 5 Conclusion

In conclusion, the present study confirmed that HLB has a favorable hepatoprotective effect, significantly improving ALD‐induced hepatic function abnormalities and hepatic fat accumulation, and effectively decreasing serum levels of AST, ALT, TG, and TC. HLB was able to improve intestinal epithelial permeability and decrease serum LPS levels, which in turn inhibited the TLR4/NF‐κB/NLRP3 pathway and attenuated the alcohol intake‐induced hepatic inflammatory response. HLB restored the composition of the intestinal flora, resulting in an increase in the relative abundance of beneficial bacteria such as Faecalibaculum, Akkermansia, and Bifidobacterium. Meanwhile, HLB restored the levels of SCFAs in the cecum contents. These results suggest that HLB combats alcohol‐induced liver injury by modulating the liver‐intestinal axis, demonstrating dual value in the field of food functionalization application and liver disease prevention, and providing a solid foundation for the development of sustainable dietary intervention strategies.

## Author Contributions

**Wen Cai:** writing – original draft, data curation, investigation, resources, software, supervision, validation. **Ru Zhang:** validation, writing – original draft. **Limin Dong:** writing – original draft, writing – review editing. **Jiayi Li:** data curation, resources, software. **Lin Wang:** data curation, resources, software. **Xuekun Shao:** data curation, resources, software. **Mingming Wang:** data curation, resources, software. **Tianyuan Jing:** data curation, resources, software. **Yanan Hu:** data curation, formal analysis, software. **Tiefeng Sun:** data curation, formal analysis, software. **Cheng Wang:** data curation, formal analysis, software. **Yi Wang:** project administration, supervision, validation, visualization. **Haitao Du:** project administration, supervision, validation, visualization. **Xiaoyan Ding:** funding acquisition, methodology, project administration, validation. **Ping Wang:** funding acquisition, project administration, resources, validation.

## Funding

This work was supported by Jinan “20 New Universities” Funding Project (202228121, 202228029), the Key R&D Project of Shandong Province (2021SFGC1205), the State Administration of Traditional Chinese Medicine Science and Technology Project (GZY‐KJS‐2023‐027), and the Shandong Traditional Chinese Medicine Science and Technology Project (Z‐2023123T, M‐2023291T).

## Ethics Statement

This study was approved by the Ethics Committee of Shandong Academy of Chinese Medicine (No. SDZYY20211001002).

## Conflicts of Interest

The authors declare no conflicts of interest.

## Supporting information


**Figure S1:** fsn371435‐sup‐0001‐FigureS1.png.

**Table S1:** fsn371435‐sup‐0002‐TableS1.docx.

**Table S2:** fsn371435‐sup‐0003‐TableS2.docx.

**Table S3:** fsn371435‐sup‐0004‐TableS3.docx.

## Data Availability

The data that supports the findings of this study are available in the Supporting Information of this article.
