# Supplementary material for: Mechanism Study on the Regulation of Intestinal Microecology by Hangover Liver‐Protecting Beverage for the Treatment of Alcoholic Liver Disease
Source: Food Sci Nutr. 2026 Jan 7;14(1):e71435. doi: 10.1002/fsn3.71435 (PMC12778415; doi:10.1002/fsn3.71435)
Supplement: Supplementary file 2 — Table S1: fsn371435‐sup‐0002‐TableS1.docx. [file FSN3-14-e71435-s003.docx]

Table S1 The Table of Primer Sequences

| Gene name | [Primer sequences](https://context.reverso.net/translation/english-chinese/Primer+sequences)（5’-3’） |
| --- | --- |
| TLR4 | TGAGGACTGGGTGAGAAATGAGC  CTGCCATGTTTGAGCAATCTCAT |
| P65 | GCAGAAAGAAGACATTGAGGTGTAT  GCGATCATCTGTGTCTGGCA |
| NLRP3 | ATGACTTTCCAGGAGTTCTTCGC  CCAAAGAGGAATCGGACAACAA |
| ASC | CAGCACAGGCAAGCACTCATT  TCATCTTGTCTTGGCTGGTGG |
| caspase-1 | AAAGACAAGCCCAAGGTGATC  CCAAGTCACAAGACCAGGCATA |
| GAPDH | CCTCGTCCCGTAGACAAAATG  TGAGGTCAATGAAGGGGTCGT |
