# Supplementary material for: Mechanism Study on the Regulation of Intestinal Microecology by Hangover Liver‐Protecting Beverage for the Treatment of Alcoholic Liver Disease
Source: Food Sci Nutr. 2026 Jan 7;14(1):e71435. doi: 10.1002/fsn3.71435 (PMC12778415; doi:10.1002/fsn3.71435)
Supplement: Supplementary file 3 — Table S2: fsn371435‐sup‐0003‐TableS2.docx. [file FSN3-14-e71435-s001.docx]

Table S2 Chemical characterization for HLB by UPLC-QTOF/MS/MS analysis.

| No. | tR  (min) | Compounds | Molecular Formula | Mode | Experiment | Error（ppm） | Fragment ions |
| --- | --- | --- | --- | --- | --- | --- | --- |
| 1 | 0.96 | Inosine | C_10_H_12_N_4_O_5_ | [M-H]^-^ | 267.0707 | -6.350 | / |
| 2 | 0.96 | Proline | C_5_H_9_NO_2_ | [M+H]^+^ | 116.0700 | -5.213 | 116.0700,70.0653 |
| 3 | 0.96 | Valine | C_5_H_11_NO_2_ | [M+H]^+^ | 118.0856 | -5.548 | 118.0856,59.0733 |
| 4 | 0.96 | L-Aspartic acid | C_4_H_8_N_2_O_3_ | [M+H]^+^ | 133.0599 | -6.528 | / |
| 5 | 1.00 | Malic Acid | C_4_H_6_O_5_ | [M-H]^-^ | 133.0124 | -5.637 | 133.0125,115.0019,89.0226,72.9914,71.0121 |
| 6 | 1.03 | Limonexic acid | C_6_H_8_O_7_ | [M-H]^-^ | 191.0179 | -3.816 | 191.0181,173.0076,129.0176,111.0070,87.0070,85.0278 |
| 7 | 1.03 | 1-Propene-1,2,3-tricarboxylic acid | C_6_H_6_O_6_ | [M-H]^-^ | 173.0074 | -3.840 | 173.0075,111.0070,85.0278 |
| 8 | 1.06 | citramalic acid | C_5_H_8_O_5_ | [M-H]^-^ | 147.0281 | -4.760 | 147.0282,129.0175,103.0381,87.0070,85.0277 |
| 9 | 1.11 | Adenine | C_5_H_5_N_5_ | [M+H]^+^ | 136.0609 | -6.407 | 136.0609,119.0346,91.0541 |
| 10 | 1.13 | Uridine | C_9_H_12_N_2_O_6_ | [M-H]^-^ | 243.0606 | -2.314 | / |
| 11 | 1.14 | 2'-Deoxyadenosine | C_10_H_13_N_5_O_3_ | [M+H]^+^ | 252.1073 | -7.203 | 252.1073,234.0933,136.0609,117.0541 |
| 12 | 1.17 | Pyroglutamic acid | C_5_H_7_NO_3_ | [M+H]^+^ | 130.0492 | -5.149 | 130.0492,84.0444,70.0653 |
| 13 | 1.17 | Guanosine | C_10_H_13_N_5_O_5_ | [M+H]^+^ | 284.0971 | -6.494 | / |
| 14 | 1.21 | D-tert-Leucine/l-isoleucine | C_6_H_13_NO_2_ | [M+H]^+^ | 132.1012 | -5.338 | 132.1013,114.0908,86.0964,69.0701 |
| 15 | 1.22 | N-Acetyl-glutamic acid | C_7_H_11_NO_5_ | [M-H]^-^ | 188.0552 | -0.792 | / |
| 16 | 1.22 | Succinic acid | C_4_H_6_O_4_ | [M-H]- | 117.0176 | -5.428 | 117.0176,99.0070,73.0278 |
| 17 | 1.35 | 2-Trimethyl citrate | C_7_H_10_O_7_ | [M-H]- | 205.0338 | -2.337 | 205.0338,143.0332,111.0070,87.0070 |
| 18 | 1.48 | phenylalanine | C_9_H_11_NO_2_ | [M+H]^+^ | 166.0852 | -6.353 | 166.0849,149.0586,131.0483,120.0801,103.0539 |
| 19 | 1.60 | 6”-O-xylosyl-glycitin/Daidzein-4',7-glucoside | C_27_H_30_O_14_ | [M-H]^-^ | 577.1544 | -1.355 | 577.1545,457.1128,429.1168,294.0525,266.0574 |
| 20 | 1.88 | Neochlorogenic acid | C_16_H_18_O_9_ | [M-H]^-^ | 353.0866 | -0.307 | 353.0865,191.0545,179.0334,135.0434 |
| 21 | 1.95 | Eugenol | C_10_H_12_O_2_ | [M-H]^-^ | 163.0746 | -4.637 | / |
| 22 | 1.99 | protocatechuic acid | C_7_H_6_O_4_ | [M-H]^-^ | 153.0177 | -3.497 | 153.0176,109.0277 |
| 23 | 2.13 | 1,5-dimethyl citrate | C_8_H_12_O_7_ | [M-H]^-^ | 219.0496 | -1.503 | 219.0490,157.0488,111.0070,87.0070 |
| 24 | 2.21 | Esculin | C_15_H_16_O_9_ | [M-H]^-^ | 339.0711 | 0.123 | / |
| 25 | 2.33 | DL-Tryptophan/(-)-Tryptophan | C_11_H_12_N_2_O_2_ | [M+H]^+^ | 205.0957 | -7.090 | 188.0693,146.0590,118.0645 |
| 26 | 2.38 | Caulophyllogenin | C_6_H_6_O_2_ | [M-H]^-^ | 109.0278 | -5.558 | 109.0277,108.0199 |
| 27 | 2.52 | trans-4-coumaroyl-3-O-Sucrose fatty acid esters | C_21_H_28_O_13_ | [M-H]^-^ | 487.1442 | -0.856 | / |
| 28 | 2.54 | vitexin | C_21_H_20_O_10_ | [M+H]^+^ | 433.1093 | -8.366 | 433.1099、415.0992、397.0887、379.0790、313.0684、295.0577、283.0582 |
| 28 | 2.56 | vitexin | C_21_H_20_O_10_ | [M-H]^-^ | 431.0964 | -2.025 | 431.0968,311.0549,283.0601 |
| 29 | 2.73 | 4-O-Coumaroylquinic acid | C_16_H_18_O_8_ | [M-H]^-^ | 337.0917 | -0.279 | 337.0916,191.0546,163.0383,119.0485 |
| 30 | 2.77 | 6-O-Caffeoylglucopyranose | C_15_H_18_O_9_ | [M-H]^-^ | 341.0865 | -0.611 | 341.0861,281.0656,251.0547,221.0440,179.0333,161.0227,135.0435 |
| 31 | 2.85 | 3'-Hydroxy Puerarin | C_21_H_20_O_10_ | [M+H]^+^ | 433.1092 | -8.597 | 433.1099,415.0992,397.0887,367.0786,337.0679,313.0684,283.0582 |
| 32 | 2.86 | 8－Deoxylactucin | C_15_H_16_O_4_ | [M+H]^+^ | 261.1109 | -4.732 | / |
| 33 | 2.91 | Chlorogenic acid | C_16_H_18_O_9_ | [M-H]^-^ | 353.0865 | -0.591 | 353.0872,191.0545 |
| 34 | 2.91 | 7-Hydroxycoumarine | C_9_H_6_O_3_ | [M-H]^-^ | 161.0227 | -3.854 | / |
| 34 | 2.93 | 7-Hydroxycoumarine | C_9_H_6_O_3_ | [M+H]^+^ | 163.0379 | -6.567 | 173.0615,163.0378,145.0275,135.0432,117.0330,107.0488,89.0385 |
| 35 | 2.95 | p-Hydroxybenzoic acid | C_7_H_6_O_3_ | [M-H]^-^ | 137.0227 | -4.529 | 137.0227,93.0329 |
| 36 | 2.95 | Protamine sulfates | C_7_H_6_O_3_ | [M-H]^-^ | 137.0227 | -4.529 | 137.0227,119.0122,93.0329 |
| 37 | 2.98 | Cichorioside B | C_21_H_28_O_10_ | [M-H]^-^ | 439.1595 | -0.850 | / |
| 38 | 3.20 | Cryptochlorogenic acid | C_16_H_18_O_9_ | [M-H]^-^ | 353.0865 | -0.591 | 353.0862,191.0545,179.0334,173.0439,135.0435 |
| 39 | 3.38 | 2-(Carboxymethyl)-4,5-Dimethoxybenzoic acid | C_11_H_12_O_6_ | [M-H]^-^ | 239.0549 | -0.479 | 239.0547,179.0333,177.0541,149.0591,133.0642,107.0485 |
| 40 | 3.58 | pediculari-lactone | C_9_H_12_O_4_ | [M+H]^+^ | 185.0800 | -4.514 | 185.0798,170.0560,153.0536,139.0380,125.0591,110.0358 |
| 41 | 3.67 | 6,7-Dihydroxycoumarin | C_9_H_6_O_4_ | [M-H]^-^ | 177.0179 | -1.893 | 199.3186,177.0177,147.0434,133.0278,119.0485,93.0329 |
| 42 | 3.69 | cis-4-coumaric acid | C_9_H_8_O_3_ | [M+H]^+^ | 165.0536 | -6.184 | 165.0536,147.0430,119.0486 |
| 43 | 3.71 | catechin | C_15_H_14_O_6_ | [M-H]^-^ | 289.0707 | -0.223 | 289.0706,245.0806,221.0806,203.0697,179.0334,125.0226,109.0277 |
| 44 | 3.98 | Caffeic acid | C_9_H_8_O_4_ | [M+H]^+^ | 181.0484 | -6.076 | 181.0485,163.0378,145.0275,135.0432 |
| 44 | 3.99 | Caffeic acid | C_9_H_8_O_4_ | [M-H]^-^ | 179.0334 | -2.710 | 179.0333,135.0434 |
| 45 | 4.15 | Puerarin | C_21_H_20_O_9_ | [M+H]^+^ | 417.1144 | -8.651 | 417.1149,399.1045,381.0942,321.0735,297.0736,267.0633 |
| 46 | 4.48 | Puerarin 6''-O-xyloside/Puerarin apioside | C_26_H_28_O_13_ | [M-H]^-^ | 547.1438 | -1.494 | 547.1442,325.0711,295.0599,267.0652 |
| 46 | 4.46 | Puerarin apioside/Puerarin 6''-O-xyloside | C_26_H_28_O_13_ | [M+H]^+^ | 549.1562 | -7.588 | 549.1564,417.1150,399.1046,321.0734,297.0735,267.0634 |
| 47 | 4.48 | Isospinosin | C_28_H_32_O_15_ | [M-H]^-^ | 607.1650 | -1.230 | 607.1650,427.1027,325.0705,297.0755,282.0522 |
| 48 | 4.59 | epicatechin | C_15_H_14_O_6_ | [M-H]^-^ | 289.0706 | -0.223 | 289.0706,245.0806,203.0697,109.0277 |
| 48 | 4.61 | epicatechin | C_15_H_14_O_6_ | [M+H]^+^ | 291.0841 | -7.608 | 291.0860,207.0637,165.0537,147.0432,139.0381,123.0435 |
| 49 | 4.80 | 3'-Methoxypuerarin/Calycosin-7-O-beta-D-glucoside | C_22_H_22_O_10_ | [M-H]^-^ | 445.1122 | -1.625 | 445.1127,325.0704,310.0471,297.0753,282.0524,268.0357 |
| 50 | 4.83 | Neoeriocitrin | C_27_H_32_O_15_ | [M-H]^-^ | 595.1650 | -1.254 | 595.1654,287.0550,259.0599,125.0227 |
| 51 | 4.94 | Ampelopsin | C_15_H_12_O_8_ | [M-H]^-^ | 319.0444 | -1.391 | 319.0451,301.0338,257.0443,215.0333,193.0127,175.0021,125.0227 |
| 52 | 4.97 | Puerarin 6''-O-xyloside/Puerarin apioside | C_26_H_28_O_13_ | [M-H]^-^ | 547.1438 | -1.494 | 547.1442,295.0600,267.0651 |
| 52 | 4.99 | Puerarin 6''-O-xyloside/Puerarin apioside | C_26_H_28013_ | [M+H]^+^ | 549.1561 | -7.588 | 549.1561,417.1147,399.1045,381.0939,321.0734,297.0734,267.0633 |
| 53 | 5.01 | Vicenin -2/Vitexin -2''-O-β-D-glucoside/Isovitexin 2''-O-β-D-glucoside | C_27_H_30_O_15_ | [M-H]^-^ | 593.1494 | -1.174 | 593.1497,473.1077,383.0759,353.0652,297.0758 |
| 54 | 5.28 | Nicotiflorin/Apigenin 7-O-D-Glucopyranose, 1-[3-(3,4-dihydroxyphenyl)-2-propenoate] | C_27_H_30_O_15_ | [M+H]^+^ | 595.1629 | -4.783 | / |
| 55 | 5.28 | Lactucine | C_15_H_16_O_5_ | [M+H]^+^ | 277.1052 | -6.677 | 277.1050,259.0952,241.0840,213.0892,195.0789,185.0949 |
| 56 | 5.66 | 6”-O-xylosyl-glycitin/Daidzein-4',7-glucoside | C_27_H_30_O_14_ | [M-H]^-^ | 577.1549 | -0.488 | 577.1549,531.2433,325.0704,297.0757,282.0522 |
| 57 | 5.69 | Citronellol-β-D-glucopyranoside | C_15_H_20_O_6_ | [M-H]^-^ | 295.1175 | -0.389 | 295.1176,251.1275,207.0648,192.0412,135.0434 |
| 58 | 6.02 | 3-O-Caffeoylquinic acid methyl ester/Famprofazone | C_17_H_20_O_9_ | [M-H]^-^ | 367.1024 | -0.113 | 367.1032,329.8421,191.0546,173.0439 |
| 59 | 6.29 | Jasmonic acid | C_12_H_18_O_4_ | [M+H]^+^ | 227.1261 | -7.421 | 227.1261,209.1157,191.1053,167.1055,149.0951,131.0848,85.0648 |
| 60 | 6.35 | UNII:319R5C7293 | C_10_H_16_O | [M-H]^-^ | 151.1111 | -4.246 | / |
| 61 | 6.60 | Swertisin | C_22_H_22_O_11_ | [M-H]^-^ | 461.1073 | -1.166 | 415.1022,253.0493 |
| 62 | 6.81 | 7-[[2-O-(6-deoxy-alpha-L-mannopyranosyl)-beta-D-glucopyranosyl]oxy]-3,5-dihydroxy-2-(4-hydroxyphenyl)-4H-benzopyran-4-one | C_27_H_30_O_15_ | [M-H]^-^ | 593.1501 | -0.006 | / |
| 63 | 7.47 | Vanillic acid | C_8_H_8_O_4_ | [M-H]^-^ | 167.0335 | -2.306 | / |
| 64 | 7.61 | 3,5-di-O-galloylshikimic acid | C_21_H_18_O_13_ | [M-H]^-^ | 477.0663 | -0.140 | 477.0649,301.0341 |
| 65 | 8.27 | Chicoric acid | C_22_H_18_O_12_ | [M-H]^-^ | 473.0714 | -0.111 | / |
| 66 | 8.53 | genistein-8-c-glucoside | C_26_H_28_O_14_ | [M-H]^-^ | 563.1388 | -1.300 | 563.1385,341.0645,311.0548,283.0600 |
| 67 | 8.56 | Vitexin-2''-O-rhamnoside | C_27_H_30_O_14_ | [M-H]^-^ | 577.1543 | -1.528 | / |
| 68 | 8.72 | Kaempferol | C_15_H_10_O_6_ | [M+H]^+^ | 287.0529 | -0.732 | 287.0528、279.8990、153.0170、137.0223 |
| 69 | 8.74 | kaempferol 3-O-glucoside | C_21_H_20_O_11_ | [M-H]^-^ | 447.0922 | 0.027 | 447.0919,285.0394 |
| 70 | 8.76 | Luteolin | C_15_H_10_O_6_ | [M+H]^+^ | 287.0529 | -7.366 | 287.0528,279.8990 |
| 71 | 8.93 | Naringin | C_27_H_32_O_14_ | [M-H]^-^ | 579.1709 | 0.118 | 579.1693,480.5948,449.4832,271.0605,151.0019 |
| 72 | 9.00 | 5,7-Dihydroxy-3',4',5'-trimethoxyflavone | C_18_H_16_O_7_ | [M-H]- | 343.0827 | 4.287 | / |
| 73 | 9.36 | Taxifolin | C_15_H_12_O_7_ | [M-H]- | 303.0496 | -1.086 | 303.0482,285.0392,125.0227 |
| 74 | 9.40 | Vicenin -2/Vitexin -2''-O-β-D-glucoside/Isovitexin 2''-O-β-D-glucoside | C_27_H_30_O_15_ | [M-H]- | 593.1500 | -0.163 | 593.1489,505.8198,449.2050,341.0650326.0442,298.0468 |
| 75 | 9.61 | Vicenin -2/Vitexin -2''-O-β-D-glucoside/Isovitexin 2''-O-β-D-glucoside | C_27_H_30_O_15_ | [M-H]^-^ | 593.1524 | 3.884 | 593.1469,442.0757,413.0901,293.0464 |
| 76 | 9.65 | Laricitrin | C_16_H_12_O_8_ | [M-H]^-^ | 331.0461 | 3.795 | 331.0455,300.0272,271.0248,178.9978,151.0025 |
| 77 | 9.65 | Ellagic acid | C_14_H_6_O_8_ | [M-H]^-^ | 300.9980 | 0.354 | / |
| 78 | 9.72 | Hovenitins I/Hovenitins II | C_16_H_14_O_8_ | [M-H]^-^ | 333.0606 | 0.319 | 333.0607,315.0501,207.0285,178.9964,125.0227 |
| 79 | 9.74 | Hovenitin II | C_16_H_14_O_8_ | [M+H]^+^ | 335.0738 | -6.995 | 335.0724,371.0632,289.0684,195.0274,153.0173 |
| 80 | 10.02 | Pueroside A | C_29_H_34_O_14_ | [M-H]^-^ | 605.1861 | -0.631 | 605.1859,297.0755,253.0857 |
| 81 | 10.02 | 3'-Methoxydaidzein | C_16_H_12_O_5_ | [M+HCOO]^-^ | 329.0643 | -3.887 | / |
| 82 | 10.34 | ferulic acid | C_10_H_10_O_4_ | [M-H]^-^ | 193.0491 | -2.255 | 193.0490,178.0255,134.0356,101.9231 |
| 83 | 11.03 | Isoquercitrin | C_21_H_20_O_12_ | [M-H]^-^ | 463.0871 | -0.221 | / |
| 84 | 11.24 | Spinosin | C_28_H_32_O_15_ | [M-H]^-^ | 607.1659 | 0.253 | / |
| 85 | 11.28 | Luteolin-7-O-glucuronide | C_21_H_18_O_12_ | [M-H]^-^ | 461.0712 | -0.547 | 461.0712,285.0394,113.0227 |
| 86 | 11.44 | Luteolin-7-O-glucoside | C_21_H_20_O_11_ | [M+H]^+^ | 449.1076 | -0.530 | 287.0527,199.6203 |
| 87 | 11.44 | Emodin | C_15_H_10_O_5_ | [M+H]^+^ | 271.0580 | -7.747 | 271.0580,215.0686,153.0170 |
| 88 | 11.46 | Isorhamnetin-3-O-beta-D-Glucoside | C_22_H_22_O_12_ | [M-H]^-^ | 477.1026 | -0.319 | / |
| 89 | 11.48 | Genistein | C_15_H_10_O_5_ | [M+H]^+^ | 271.0580 | -7.747 | 271.0580,243.0634,215.0686,153.0170 |
| 90 | 12.63 | Coumarin | C_9_H_6_O_2_ | [M-H]^-^ | 145.0279 | -3.489 | 145.0279,117.0328 |
| 91 | 12.63 | Phloretin 3',5'-Di-C-glucoside | C_27_H_34_O_15_ | [M-H]^-^ | 597.1813 | -0.162 | / |
| 92 | 13.06 | Ononin | C_22_H_22_O_9_ | [M-H]^-^ | 429.1177 | -0.719 | 448.6528,429.1176,309.0757,291.0647,281.0808,266.0575 |
| 93 | 13.36 | Pueroside B | C_30_H_36_O_15_ | [M-H]^-^ | 635.1965 | -0.861 | 616.6561,473.1441,311.0916,267.1017,252.0783 |
| 94 | 13.72 | (+)-Eriodictyol | C_15_H_12_O_6_ | [M-H]^-^ | 287.0545 | -1.792 | / |
| 95 | 13.74 | Ononin | C_22_H_22_O_9_ | [M+H]^+^ | 431.1320 | -3.847 | 431.1307,311.0890,281.0788 |
| 96 | 13.79 | Daidzein | C_15_H_10_O_4_ | [M-H]^-^ | 253.0491 | -1.720 | 253.0493 |
| 97 | 14.23 | Isochlorogenic acid C(4,5) | C_25_H_24_O_12_ | [M-H]^-^ | 515.1180 | -0.781 | / |
| 98 | 14.26 | Kaempferol-3-beta-O-glucuronide | C_21_H_18_O_12_ | [M-H]^-^ | 461.0713 | -0.330 | / |
| 99 | 14.68 | Quercitrin | C_21_H_20_O_11_ | [M+H]^+^ | 449.1099 | 4.592 | / |
| 100 | 15.42 | Apigenin-7-glucuronide | C_21_H_18_O_11_ | [M-H]^-^ | 445.0763 | -0.534 | 445.0766,269.0446,175.0231,113.0227,85.0278 |
| 101 | 15.81 | threo-dihydroxydehydrodiconiferyl alcohol | C_20_H_24_O_8_ | [M+HCOO]^-^ | 437.1442 | -0.053 | 437.1449,325.0911,265.0709,235.0598,205.049,163.0385,145.0279,119.0484 |
| 102 | 16.30 | Cosmosiin | C_21_H_20_O_10_ | [M+H]^+^ | 433.1113 | -3.748 | / |
| 103 | 16.45 | delphinidin | C_15_H_11_O_7_ | [M-H]^-^ | 302.0400 | -6.966 | / |
| 104 | 17.07 | Kaempferol--3-O-(6″-O-acetyl)-β-glucosidase | C_23_H_22_O_12_ | [M-H]^-^ | 489.1029 | 0.302 | / |
| 105 | 17.96 | Formononetin | C_16_H_12_O_4_ | [M-H]^-^ | 267.0653 | 0.429 | 267.0653,252.0415,119.0484 |
| 106 | 18.09 | Ononin | C_22_H_22_O_9_ | [M+H]^+^ | 431.1307 | -6.863 | 431.1310,269.0789 |
| 107 | 21.76 | 11beta,13-Dihydrolactucin | [C_23_H_24_O_7_](https://www.chembk.com/cn/search/C23H24O7) | [M-H]^-^ | 411.1436 | -0.558 | / |
| 108 | 22.14 | Formononetin | C_16_H_12_O_4_ | [M-H]^-^ | 267.0650 | -0.694 | 267.0653,252.0416 |
| 109 | 22.20 | Corosolic acid | C_30_H_48_O_4_ | [M+H]^+^ | 473.3620 | -1.133 | / |
| 110 | 22.20 | Oleanic acid | C_30_H_48_O_3_ | [M+H]^+^ | 457.3644 | -7.044 | 457.3663,439.3546,411.3554,295.5161,109.1007,95.0854 |
| 111 | 22.31 | Ursolic Acid | C_30_H_48_O_3_ | [M+H]^+^ | 457.3647 | -6.388 | 457.3663,439.3546,163.1474,135.1164,109.1007 |
| 112 | 22.34 | Quercetin | C_15_H_10_O_7_ | [M+H]^+^ | 303.0484 | -5.046 | 303.0475,285.0371,257.0428,229.0479,153.0170,137.0225 |
| 113 | 22.34 | Ganoderol A | C_30_H_46_O_2_ | [M+H]^+^ | 439.3542 | -6.503 | 439.3568,421.3446,299.2346 |
| 114 | 22.36 | HoveninA | C_26_H_24_O_12_ | [M-H]^-^ | 527.1185 | 0.185 | / |
| 115 | 22.42 | Glochidone | C_30_H_46_O | [M+H]^+^ | 423.3595 | -6.242 | 423.3612,293.6471,269.2243,201.1633,95.0855 |
| 116 | 22.45 | Emmolic Acid | C_30_H_46_O_5_ | [M+H]^+^ | 487.3389 | -5.953 | 487.3429,469.3260,329.6899,95.0855 |
| 117 | 22.47 | Lactupicrin | C_23_H_22_O_7_ | [M-H]^-^ | 409.1282 | 0.050 | / |
| 118 | 22.51 | Apigenin | C_15_H_10_O_5_ | [M-H]^-^ | 269.0447 | 0.930 | / |
| 119 | 22.65 | arjungenin | C_30_H_48_O_6_ | [M-H]^-^ | 503.3363 | -0.826 | / |
| 120 | 22.77 | Naringenin | C_15_H_12_O_5_ | [M+H]^+^ | 273.0753 | -1.648 | 273.0739,171.0277,153.0171,147.0431 |
| 121 | 25.28 | Betulonic acid | C_30_H_46_O_3_ | [M+H]^+^ | 455.3527 | 1.599 | 455.3503,437.3378,330.1386,231.1780,215.1780,189.1621 |
