# Supplementary material for: Mechanism Study on the Regulation of Intestinal Microecology by Hangover Liver‐Protecting Beverage for the Treatment of Alcoholic Liver Disease
Source: Food Sci Nutr. 2026 Jan 7;14(1):e71435. doi: 10.1002/fsn3.71435 (PMC12778415; doi:10.1002/fsn3.71435)
Supplement: Supplementary file 4 — Table S3: fsn371435‐sup‐0004‐TableS3.docx. [file FSN3-14-e71435-s004.docx]

Table S3 Results of the analysis of the binding of the main active ingredient and TLR4.

|  | Molecule | Types of Bonds | Residues | Binding Energy  (kcal/mol) |
| --- | --- | --- | --- | --- |
| TLR4 | Epicatechin | Hydrogen Bond | LYS-59、VAL-72、PRO-124、TRP-180 | -7.4 |
|  |  | Hydrophobic Interaction | LEU-56、TYR-229、PRO-33、PHE-34 |  |
|  | Quercetin | Hydrogen Bond | LYS-59、VAL-72、ERP-180 | -7.4 |
|  |  | Hydrophobic Interaction | LEU-56、PHE-34、PRO-33、VAL-127 |  |
|  | Ampelopsin | Hydrogen Bond | LYS-59、VAL-72、TRP-180、PRO-124 | -7.3 |
|  |  | Hydrophobic Interaction | PRO-33、PHE-34 |  |
|  | Catechin | Hydrogen Bond | LYS-59、VAL-72、TRP-180、PRO-124 | -7.2 |
|  |  | Hydrophobic Interaction | LEU-56、PHE-34、PRO-33、TYR-299 |  |
|  | Kaempferol | Hydrogen Bond | LYS-59、PRO-124、GLY-128 | -7 |
|  |  | Hydrophobic Interaction | LEU-71、PHE-34、LEU-56 |  |
|  | Naringenin | Hydrogen Bond | LYS-59、TRP-180 | -7 |
|  |  | Hydrophobic Interaction | PRO-33、PHE-34、TYR-229、LEU-56、LEU-71 |  |
|  | Ferulic acid | Hydrophobic Interaction | TYR-229、LEU-226、PHE-225 | -5.7 |
|  | Caffeic acid | Hydrogen Bond | GYL-128 | -5.5 |
|  |  | Hydrophobic Interaction | TYR-229、LEU-226、PHE-225 |  |
|  | Protocatechuic acid | Hydrogen Bond | ARG-183、TRP-222 | -5.3 |
|  |  | Hydrophobic Interaction | TYR-229、TRP-222 |  |
